# Redox Regulation of Cardiac ASK1 (Apoptosis Signal-Regulating Kinase 1) Controls p38-MAPK (Mitogen-Activated Protein Kinase) and Orchestrates Cardiac Remodeling to Hypertension

**DOI:** 10.1161/HYPERTENSIONAHA.119.14556

**Published:** 2020-09-09

**Authors:** Daniel N. Meijles, Joshua J. Cull, Thomais Markou, Susanna T.E. Cooper, Zoe H.R. Haines, Stephen J. Fuller, Peter O’Gara, Mary N. Sheppard, Sian E. Harding, Peter H. Sugden, Angela Clerk

**Affiliations:** 1 From the Molecular and Clinical Sciences Institute (D.N.M., S.T.E.C., Z.H.R.H.), St George’s University of London, United Kingdom; 2 CRY Cardiovascular Pathology Department (M.N.S.), St George’s University of London, United Kingdom; 3 School of Biological Sciences, University of Reading, United Kingdom (D.N.M., J.J.C., T.M., S.J.F., P.H.S., A.C.), St. George’s Healthcare NHS Trust, London, United Kingdom; 4 National Heart and Lung Institute, Faculty of Medicine, Imperial College London, United Kingdom (P.O., S.E.H.).

**Keywords:** angiotensin, heart disease, hypertension, oxidative stress reactive oxygen species, protein kinase

## Abstract

Systemic hypertension increases cardiac workload causing cardiomyocyte hypertrophy and increased cardiac fibrosis. An underlying feature is increased production of reactive oxygen species. Redox-sensitive ASK1 (apoptosis signal-regulating kinase 1) activates stress-regulated protein kinases (p38-MAPK [mitogen-activated protein kinases] and JNKs [c-Jun N-terminal kinases]) and promotes fibrosis in various tissues. Here, we determined the specificity of ASK1 signaling in the heart, with the hypothesis that ASK1 inhibitors may be used to manage fibrosis in hypertensive heart disease. Using immunoblotting, we established that moderate levels of H_2_O_2_ activate ASK1 in neonatal rat cardiomyocytes and perfused rat hearts. ASK1 was activated during ischemia in adult rat hearts, but not on reperfusion, consistent with activation by moderate (not high) reactive oxygen species levels. In contrast, IL (interleukin)-1β activated an alternative kinase, TAK1 (transforming growth factor–activated kinase 1). ASK1 was not activated by IL1β in cardiomyocytes and activation in perfused hearts was due to increased reactive oxygen species. Selonsertib (ASK1 inhibitor) prevented activation of p38-MAPKs (but not JNKs) by oxidative stresses in cultured cardiomyocytes and perfused hearts. In vivo (C57Bl/6J mice with osmotic minipumps for drug delivery), selonsertib (4 mg/[kg·d]) alone did not affect cardiac function/dimensions (assessed by echocardiography). However, it suppressed hypertension-induced cardiac hypertrophy resulting from angiotensin II (0.8 mg/[kg·d], 7d), with inhibition of *Nppa/Nppb* mRNA upregulation, reduced cardiomyocyte hypertrophy and, notably, significant reductions in interstitial and perivascular fibrosis. Our data identify a specific reactive oxygen species→ASK1→p38-MAPK pathway in the heart and establish that ASK1 inhibitors protect the heart from hypertension-induced cardiac remodeling. Thus, targeting the ASK1→p38-MAPK nexus has potential therapeutic viability as a treatment for hypertensive heart disease.

## Abstract 2

Supplemental Digital Content is available in the text.

Hypertension contributes significantly to heart failure, thus being a prominent cause of morbidity and mortality worldwide.^1^ Despite its prevalence, viable treatments for hypertensive heart failure remain limited. Initially, the heart adapts to the increased workload resulting from hypertension to maintain cardiac output.^2^ This is achieved in part by hypertrophy of terminally differentiated contractile cardiomyocytes, with associated increases in size and myofibrillar content, along with adaptation of the myofibrillar apparatus. This process is unsustainable in the long term, resulting in heart failure. Pathological changes include increased cardiomyocyte cell death,^3^ capillary rarefaction,^4^ myocardial inflammation, and increased fibrosis.^5^ Developing strategies to control each of these is necessary for management of hypertensive heart failure,^6^ but reducing fibrosis is likely to be particularly important in reducing workload on cardiomyocytes and increasing myocyte survival, facilitating angiogenesis, and reducing inflammation.

The p38 (p38-MAPK [mitogen-activated protein kinases]) and JNK (c-Jun N-terminal kinases) cascades are key modulators of the cell survival versus apoptosis/death balance. Both are implicated in the development of heart failure,^7^ being activated in cardiomyocytes and the heart by pathophysiological stresses such as redox stress and proinflammatory cytokines, including IL (interleukin)-1β.^8^ p38-MAPKs and JNKs are generally activated in parallel, the only well-documented exception being with ischemia where there is selective activation of p38-MAPKs.^9^ Hypertension is associated with increased redox stress and some degree of inflammation,^10^ so modulating p38-MAPK or JNK activities may be therapeutically beneficial in hypertensive heart failure. p38-MAPKs and JNKs are, of themselves, potential therapeutic targets but, because of their role in inflammation,^11^ it may be more desirable to target specific inputs into the cascade to modulate activity rather than block the signal completely. p38-MAPKs and JNKs are phosphorylated/activated by upstream MKKs (MAPK kinases), MKK3/6, and MKK4/7, respectively. These are phosphorylated/activated by upstream MAP3Ks (MKK kinases) which are more numerous, potentially responding selectively to specific stresses.^12^ The greater diversity at the MAP3K level provides increased potential for selective targeting. Two such kinases are *MAP3K5* (ASK1 [apoptosis signal-regulating kinase 1]) and *MAP3K7* (TAK1 [transforming growth factor–activated kinase 1]), each of which has been placed upstream of both p38-MAPKs and JNKs in noncardiac cells where they regulate cell death responses.^13^

ASK1 is activated by myriad cues that alter the cellular redox balance.^14,15^ ASK1 is inhibited by association with thioredoxin, oxidation of which (by elevated ROS [reactive oxygen species], such as H_2_O_2_) induces complex dissociation and ASK1 autophosphorylation of the activation loop (Thr838 in humans; Thr845 in mice/rats). ASK1 is associated with development of fibrosis in various tissues and is a therapeutic target for fibrotic diseases, including pulmonary arterial hypertension, chronic kidney disease, and nonalcoholic steatohepatitis. Indeed, ASK1 inhibitors have been developed and passed into phase-III clinical trials.^16,17^ In the heart, ASK1 is activated in mouse models of pressure-overload,^14^ ischemia/reperfusion,^18^ myocardial infarction,^14^ and hypertension induced by Ang II (angiotensin II),^19^ all of which are associated with increased ROS. Furthermore, studies in global ASK1 knockout mice demonstrate reduced cardiac cell death and remodeling in models of myocardial infarction,^20^ indicating that it plays a detrimental role. How ASK1 might be involved in cardiac hypertrophy and remodeling is still far from clear. Nevertheless, therapies targeting ASK1 are in development,^21^ and cardiac ASK1 is an attractive target for heart failure.^22^

Here, we addressed the hypothesis that (since hypertension is associated with hypoxia and ROS) ASK1 is likely to be a prominent cardiac MAP3K in hypertension, and (because ASK1 promotes fibrosis in other tissues) its inhibition potentially reduces cardiac fibrosis. With reports that ASK1 is activated by various stimuli and signals nonselectively to p38-MAPKs and JNKs, we first clarified and delineated the ASK1 signaling pathway in the heart, establishing that ASK1 was specifically and selectively activated by moderate levels of redox stress, signaling selectively to p38-MAPK (not JNKs). ASK1, therefore, has an appropriate profile for cardiac activation in hypertension where, given its profibrotic effects in other tissues, it may promote cardiac fibrosis. Consistent with this, selonsertib (GS-4997), an ASK1 inhibitor developed as an antifibrotic agent for nonalcoholic steatohepatitis,^16^ reduced cardiac fibrosis, and remodeling in mice treated with Ang II. Thus, ASK1 inhibitors represent a viable therapeutic modality for fibrosis in hypertensive heart disease.

## Methodology

See the Data Supplement for a full description of materials and methods. Descriptions of cell/animal experiments are provided below. Data from this study are available from the corresponding authors upon reasonable request.

Neonatal rat ventricular myocytes were prepared and adult rat hearts perfused as described previously.^8,23,24^ Cells were exposed to H_2_O_2_ or IL1β at the concentrations/times indicated. In some experiments, cells were preincubated with selonsertib before treatment with H_2_O_2_ or IL-1β. Hearts were equilibrated (15 minutes) and then perfused with H_2_O_2_ or IL1β, or subjected to global ischemia with/without reperfusion. In some experiments, hearts were perfused with/without selonsertib or N-acetyl cysteine during the equilibration phase. Control hearts were perfused for the same total duration as the experimental conditions.

An in vivo model of Ang II–induced hypertension (0.8 mg/[kg·d], 7d) was used to assess the effects of selonsertib (4 mg/[kg·d]) on cardiac remodeling in male wild-type C57BL/6J (10–12 week) mice. In vivo echocardiography was performed using a Vevo2100 system (Visualsonics). At the end of the experiment, mouse hearts were either perfusion fixed in situ using 10% buffered formalin and embedded for sectioning and histology, or rapidly excised and pulverized in liquid nitrogen for biochemical analyses. The use of all animals in our studies was performed in accordance with UK Animals (Scientific Procedures) Act of 1986 and the European Community Directive 86/609/EEC for animal experiments.

### Statistics

Data are presented as detailed in the figure legends. Data were collected and curated in Microsoft Excel. Statistical analysis used GraphPad Prism 8.0 with *t* tests, 1-way or 2-way ANOVA with Holm-Sidak multiple comparison post hoc test as indicated. *P*<0.05 was considered statistically significant.

## Results

### Activation of Cardiac ASK1 Is ROS-Dependent

**Figure 1.**
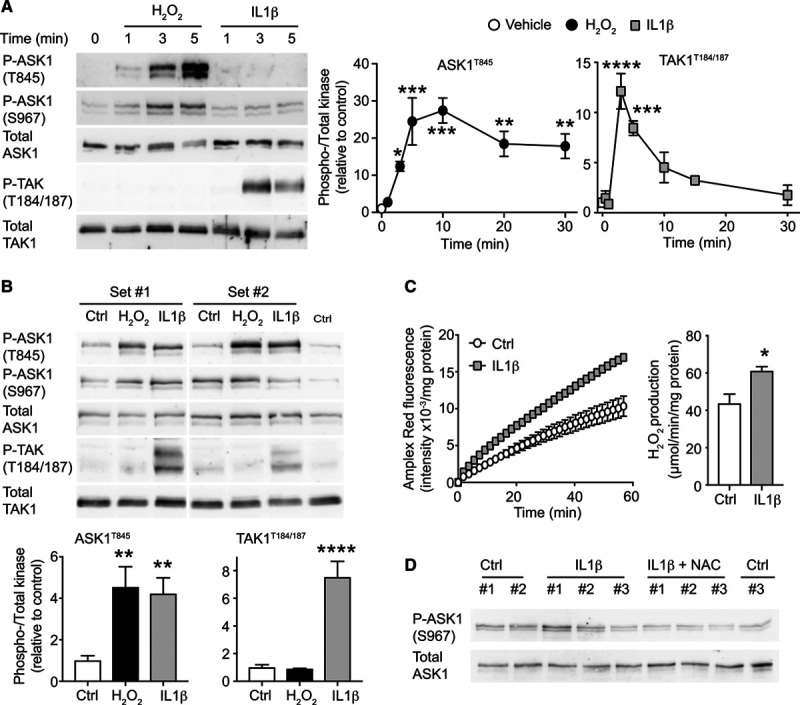
ASK1 (apoptosis signal-regulating kinase 1) is activated by oxidative stress, whereas TAK1 (transforming growth factor–activated kinase 1) is activated by IL (interleukin)-1β. **A**, Rat neonatal cardiomyocytes were exposed to 1 mmol/L H_2_O_2_ or 25 ng/mL IL-1β and samples immunoblotted for phospho (p) or total ASK1 or TAK1. **Left**, Representative blots for the acute phase (up to 5 min). **Right**, Densitometric analysis. Results are means±SEM (n=3). *P<0.05, **P<0.01, ***P<0.001, ****P<0.0001 vs vehicle (1-way ANOVA with Holm-Sidak post-test). **B**, Adult rat hearts were perfused with control (Ctrl), H_2_O_2_ (1 mmol/L, 5 min) or IL-1β (25 ng/mL, 15 min) and samples immunoblotted for phospho (p) or total kinases. **Left**, Representative immunoblots. **Right**, Densitometric analysis. Results are means±SEM (n=4). **P<0.01, ****P<0.0001 vs controls (one-way ANOVA with Holm-Sidak post-test). **C**, IL-1β (25 ng/mL, 15 min) increased H_2_O_2_ generation in adult rat perfused hearts assessed by Amplex Red. **Left**, Kinetic analysis of catalase-inhibitable fluorescence intensity. **Right**, Quantification of H_2_O_2_ production. Results are means±SEM (n=4). *P<0.05 vs vehicle (unpaired *t* test). **D**, IL-1β (25 ng/mL, 15 min) induced ASK1 activation is reduced by the reactive oxygen species scavenger N-acetyl cysteine (NAC, 4 mmol/L). Results show representative immunoblots from 3 independent samples.

Our hypothesis is that pathophysiological stresses signal through specific MAP3Ks for differential activation of p38-MAPKs versus JNKs. ASK1 is expressed in all cell types in the heart and an established ROS-sensing kinase, whereas IL-1β signals through a receptor complex, so these stimuli likely use different MAP3Ks. Activation of ASK1 compared with an alternative MAP3K (TAK1) was assessed by immunoblotting with antibodies to the activation loop phosphorylation sites. In neonatal rat cardiomyocytes, IL-1β activated TAK1, but not ASK1, whereas H_2_O_2_ activated ASK1, but not TAK1 (Figure 1A). Thus, different stimuli signal through different MAP3Ks. In perfused hearts, H_2_O_2_ activated ASK1, not TAK1, consistent with the cardiomyocyte response, but IL-1β activated both kinases (Figure 1B). We hypothesized that IL-1β may activate ASK1 via increased ROS in this context. Indeed, IL-1β significantly increased H_2_O_2_ generation in perfused hearts (Figure 1C), and perfusion of hearts with the ROS/H_2_O_2_ scavenger, N-acetyl cysteine, appeared to reduce IL-1β–induced ASK1 activation (Figure 1D) suggesting this was, indeed, the case. We conclude that ASK1 is selectively activated by ROS (which does not activate TAK1), whereas IL-1β signals more specifically through TAK1.

### Cardiac ASK1 Signals Selectively to p38-MAPKs, not JNKs

**Figure 2.**
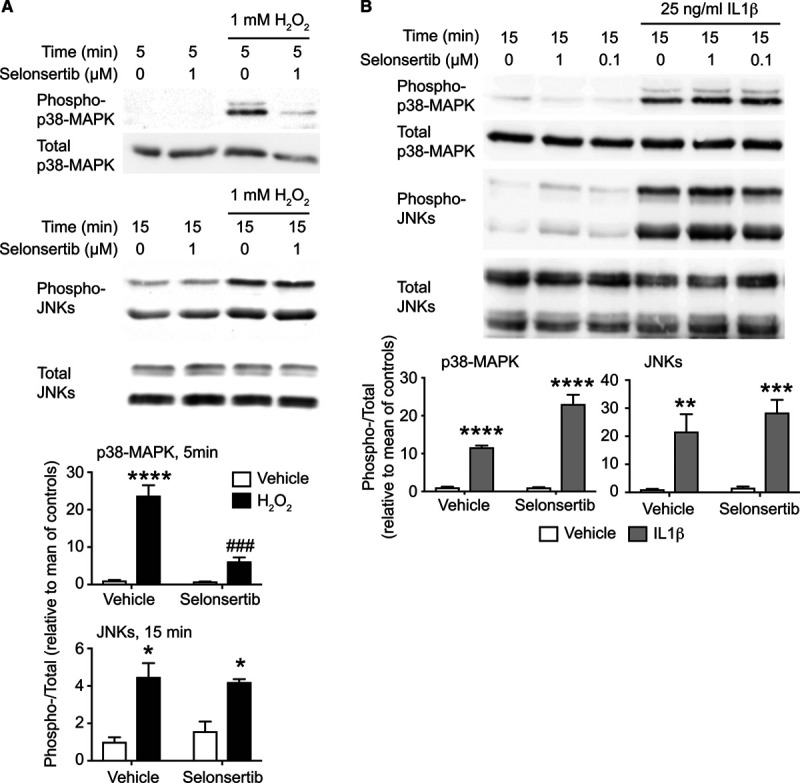
Cardiomyocyte ASK1 (apoptosis signal-regulating kinase 1) selectively signals to p38-MAPK (mitogen-activated protein kinases). Rat neonatal cardiomyocytes were exposed to 1 mmol/L H_2_O_2_ with/without 1 µM selonsertib (**A**) or to IL (interleukin)-1β (25 ng/mL, 15 min) with/without 1 or 0.1 µmol/L selonsertib (**B**). Samples were immunoblotted for phospho or total p38-MAPKs or JNKs (c-Jun N-terminal kinases). Selonsertib selectively inhibited phosphorylation of p38-MAPKs by H_2_O_2_ (**A**) but not IL-1β (**B**). **Upper**, representative immunoblots. **Lower**, Densitometric analysis. Results are means±SEM for n=4 (**A**) or n=3 (**B**). *P<0.05, **P<0.01, ***P<0.001 vs vehicle; ###*P*<0.001 vs H_2_O_2_ (1-way ANOVA with Holm-Sidak post-test).

ASK1 is reported to promote activation of both p38-MAPKs and JNKs.^14,15^ We used a selective ASK1 inhibitor, selonsertib (GS-4997),^25^ to dissect the signaling pathway in cardiomyocytes (Figure 2) and human cardiac fibroblasts (Figure S1). Selonsertib is highly selective for ASK1 (K_d_ 4.1 nmol/L) with just 2 off-target kinases (DYRK1A, K_d_ 220 nmol/L; ribosomal protein S6 kinase 4, K_d_ 430 nmol/L) that may be inhibited at higher drug concentrations,^16^ neither of which is expressed at significant levels in adult hearts.^26^ H_2_O_2_ (1 mmol/L) activated p38-MAPK in rat neonatal cardiomyocytes within 5 minutes, whereas activation of JNKs was more pronounced after 15 minutes. Pretreatment with selonsertib inhibited activation of p38-MAPK(s) but not JNKs (Figure 2A). Consistent with the IL-1β signal being mediated through an alternative pathway (Figure S2), selonsertib did not affect activation of p38-MAPKs or JNKs by IL-1β (Figure 2B). In fibroblasts, H_2_O_2_ (maximal at 100 µmol/L; Figure S1A) and IL-1β (maximal at 100 ng/ml; Figure S1B) activated p38-MAPKs, but selonsertib only inhibited activation by H_2_O_2_ (Figure S1C). Thus, cardiac ASK1 signals selectively to p38-MAPKs in the context of redox signaling.

**Figure 3.**
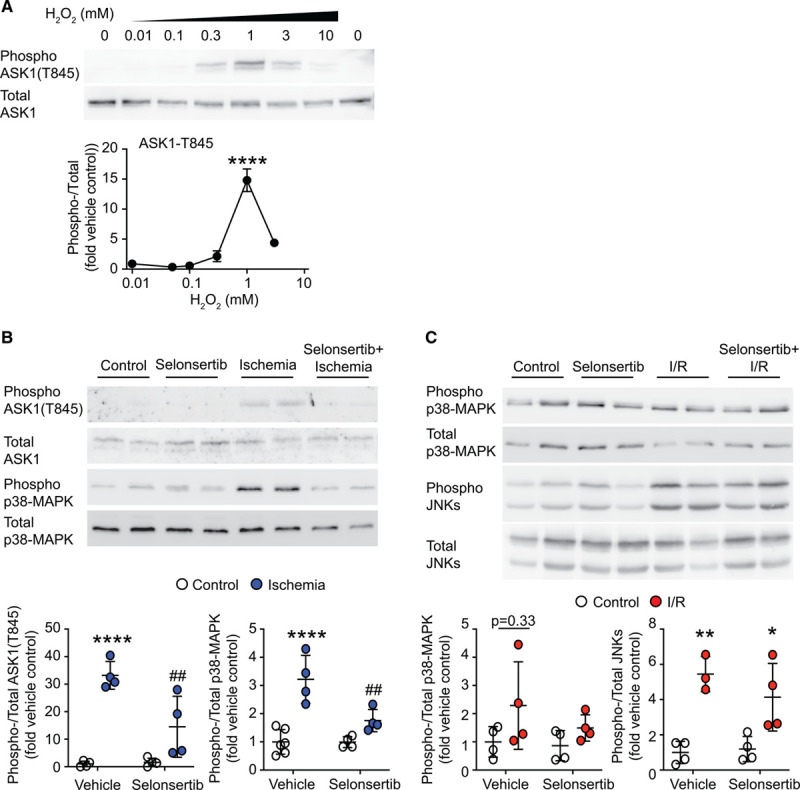
Cardiac ASK1 (apoptosis signal-regulating kinase 1) and p38-MAPKs (mitogen-activated protein kinases) are activated by moderate levels of reactive oxygen species. **A**, Rat neonatal cardiomyocytes were exposed to the indicated concentrations of H_2_O_2_ (5 min) and samples immunoblotted for phospho or total ASK1. **Upper**, Representative immunoblots. **Lower**, Densitometric analysis. Results are means±SEM (n=3). ****P<0.0001 vs vehicle (1-way ANOVA with Holm-Sidak post-test). **B** and **C**, Adult rat hearts were perfused under control conditions or subjected to ischemia (15 min) alone (**B**) or with reperfusion (I/R; **C**) with/without 1 µmol/L selonsertib. Samples were immunoblotted for phospho or total ASK1, p38-MAPKs, or JNKs (c-Jun N-terminal kinases). **Upper**, Representative immunoblots. **Lower**, Densitometric analysis. Individual data points shown with means±SEM (n=4). *P<0.05, **P<0.01, ****P<0.0001 vs vehicle; ##*P*<0.01 vs ischemia (2-way ANOVA with Holm-Sidak post-test).

The concentration-dependence of ASK1 activation by H_2_O_2_ in cardiomyocytes was bell-shaped, with activation across a range of concentrations (0.1–10 mmol/L), maximal activation at 1 mmol/L (5 minutes), and reduced activation at higher concentrations (Figure 3A). H_2_O_2_ concentrations >1 mmol/L cause cell death,^27^ but the lack of activation in this study (over 5 minutes) is not due to loss of cells because total ASK1 levels remain the same. Moreover, not all kinases become nonresponsive at higher H_2_O_2_ concentrations. For example, Akt phosphorylation becomes maximal at concentrations >3 mmol/L H_2_O_2_ in cardiomyocytes over the same duration.^28^ Thus, the bell-shaped curve for ASK1 activation (Figure 3A) suggests selective activation only at moderate (proapoptotic) concentrations of H_2_O_2_. Myocardial ischemia is associated with a moderate increase in ROS and activation of p38-MAPKs but not JNKs, whereas redox stress increases substantially on reperfusion and this leads to activation of JNKs.^9,29^ Consistent with this, we detected ASK1 phosphorylation in perfused rat hearts subjected to ischemia and this phosphorylation, together with activation of p38-MAPKs, was suppressed by the ASK1 inhibitor, selonsertib (Figure 3B). In contrast, with ischemia and reperfusion, we did not detect phosphorylation of ASK1 (not shown) and selonsertib did not inhibit phosphorylation of either p38-MAPKs or JNKs (Figure 3C). Thus, ASK1 is a redox sensor in cardiomyocytes that responds only to moderate changes in ROS levels to mediate ROS-dependent redox signaling to p38-MAPKs.

### ASK1 Inhibition Reduces Hypertension-Induced Cardiac Hypertrophy and Fibrosis

**Figure 4.**
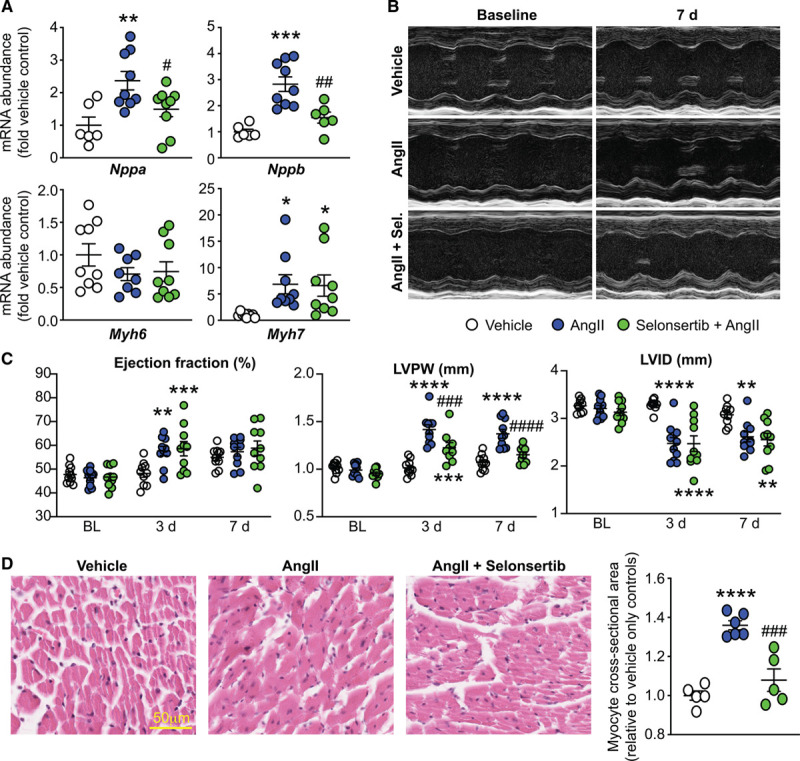
Selonsertib (Sel) inhibits Ang II (angiotensin II)–induced cardiac hypertrophy. C57Bl/6J mice were treated with 0.8 mg/(kg·d) Ang II with/without 4 mg/(kg·d) selonsertib for up to 7 d. **A**, Cardiac RNA was isolated and mRNA expression of hypertrophy-associated genes determined by quantitative polymerase chain reaction. Individual data points are shown with means±SEM. *P<0.05, **P<0.01, ***P<0.001 vs vehicle; #*P*<0.05, ##*P*<0.01 vs. Ang II (1-way ANOVA with Holm-Sidak post-test). **B** and **C**, Cardiac function and dimensions were assessed by echocardiography showing representative M-mode images of specific mice at baseline and 7 d (**B**) with quantification of echocardiograms at 3 and 7 d (**C**). Individual data points shown with means±SEM. *P<0.05, **P<0.01, ***P<0.001, ****P<0.0001 vs vehicle; ###*P*<0.001, ####*P*<0.0001 vs Ang II only (2-way repeated-measures ANOVA with Holm-Sidak post-test). **D**, Selonsertib inhibits Ang II–induced left ventricular (LV) cardiac myocyte hypertrophy assessed by hematoxylin and eosin. Scale bar: 50 µm. Individual data points shown with means±SEM. ****P<0.0001 vs vehicle; ###*P*<0.001 vs Ang II (1-way ANOVA with Holm-Sidak post-test). LVPW indicates LV posterior wall; and LVID, LV internal diameter.

Studies in global knockout mice implicate ASK1 in cardiac cell apoptosis in heart failure associated with high myocardial wall stresses (eg, myocardial infarction or pressure-overload).^20^ Knowledge of the role of cardiac ASK1 in models associated with more moderate myocardial wall stresses, such as hormone-induced hypertension, is limited. We used a model of hypertension induced by Ang II in C57Bl/6J male mice to assess this. We selected a moderate concentration that induces hypertension (0.8 mg/[kg·d] Ang II),^30^ rather than a subpressor dose (eg, 0.288 mg/[kg·d])^31^ or one more associated with sudden cardiac death (eg, >2 mg/[kg·d]). Ang II significantly increased mRNA expression of the hypertrophic markers *Nppa*, *Nppb*, and *Myh7*. Selonsertib (4 mg/[kg·d]) significantly inhibited the increase in expression of *Nppa* and *Nppb*, but not *Myh7* (Figure 4A).

We used echocardiography to assess cardiac dimensions and function at 3 days and then at 7 days (Figure 4B and 4C). Selonsertib alone did not affect any variables measured (Figure S3, Table S2). Ang II increased ejection fraction at 3 days, and this was not affected by selonsertib. At 3 and 7 days, the increase in left ventricular posterior wall thickness (but not the decrease in internal diameter) induced by Ang II in both systole (Figure 4C) and diastole were inhibited by selonsertib suggestive of a selective effect on the structure of the myocardium. Consistent with this, Ang II significantly increased left ventricular myocardial cross-sectional area compared with vehicle controls, and this was significantly inhibited by selonsertib (Figure 4D). Overall, these results demonstrate that ASK1 signaling plays a role in the cardiac hypertrophy resulting from hypertension.

**Figure 5.**
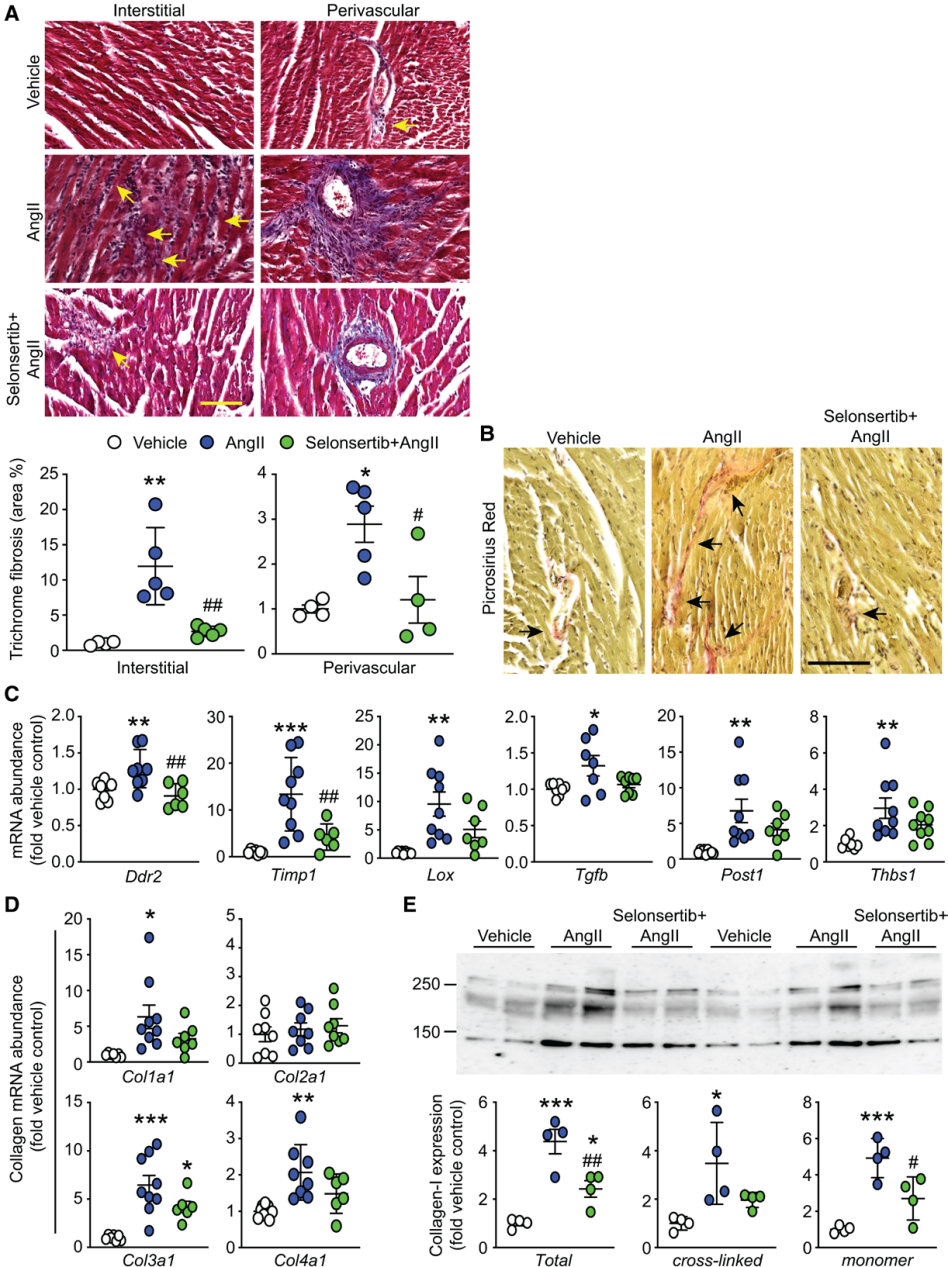
Selonsertib inhibits Ang II (angiotensin II)–induced cardiac fibrosis. C57Bl/6J mice were treated with 0.8 mg/(kg·d) Ang II with/without of 4 mg (kg·d) selonsertib for 7 d. **A**, Heart sections were stained with Trichrome showing fibrosis (blue). **Upper**, Representative images for left ventricular interstitial and perivascular regions. Scale bar: 50 µm. **Lower**, Quantitative analysis of cardiac fibrosis. Individual data points shown with means±SEM. **B**, Heart sections were stained with picrosirius red for fibrillar collagen. Scale bar: 100 µm. **C** and **D**, Cardiac RNA was prepared and mRNA expression of fibrosis and remodeling genes (**C**) or collagens (**D**) assessed by quantitative polymerase chain reaction. Individual data points shown with means±SEM. **E**, Heart samples were immunoblotted for collagen-1A1 expression. A representative blot is shown (**upper**) with densitometric analysis for total collagen 1, the cross-linked forms (upper bands) or the monomer (lower band) in the **lower** parts. *P<0.05, **P<0.01, ***P<0.001 vs vehicle; ^#^*P*<0.05, ^##^*P*<0.01 vs Ang II (1-way ANOVA with Holm-Sidak post-test).

Ang II–induced hypertension is associated with increased interstitial and perivascular fibrosis,^31^ increased expression of remodeling proteins and enzymes, and elevated levels of pathological collagens.^32^ Cardiac fibrosis was assessed in the left ventricular using Masson trichrome (for global collagen deposition; Figure 5A) and picrosirius red (for fibrillar collagens; Figure 5B). Selonsertib significantly reduced cardiac fibrosis induced by Ang II, particularly of interstitial rather than perivascular fibrosis (Figure 5A, lower). Ang II increased mRNA expression of *Ddr2* (a fibrosis marker) and *Tgfb* (that drives fibroblast activation), extracellular remodeling-related enzymes (*Timp1*, *Lox*), a profibrotic signaling molecule (*Post1*), and an extracellular matrix protein (*Thbs1*). These were all reduced in hearts from mice treated with selonsertib, although a significant inhibition was only detected for *Ddr2* and *Timp1* (Figure 5C). Ang II increased abundance of the fibrillar pathological collagens *Col1a1* and *Col3a1* and increased the basement membrane-linked *Col4a1*, with no effect on *Col2a1* (Figure 5D). As with other mRNAs assessed, selonsertib partially decreased the response to Ang II. However, selonsertib significantly reduced collagen 1 protein expression induced by Ang II (Figure 5E). These molecular findings are in accord with the reduction in cardiac fibrosis resulting from selonsertib treatment of Ang II–induced hypertension detected by histology (Figure 5A and 5B).

**Figure 6.**
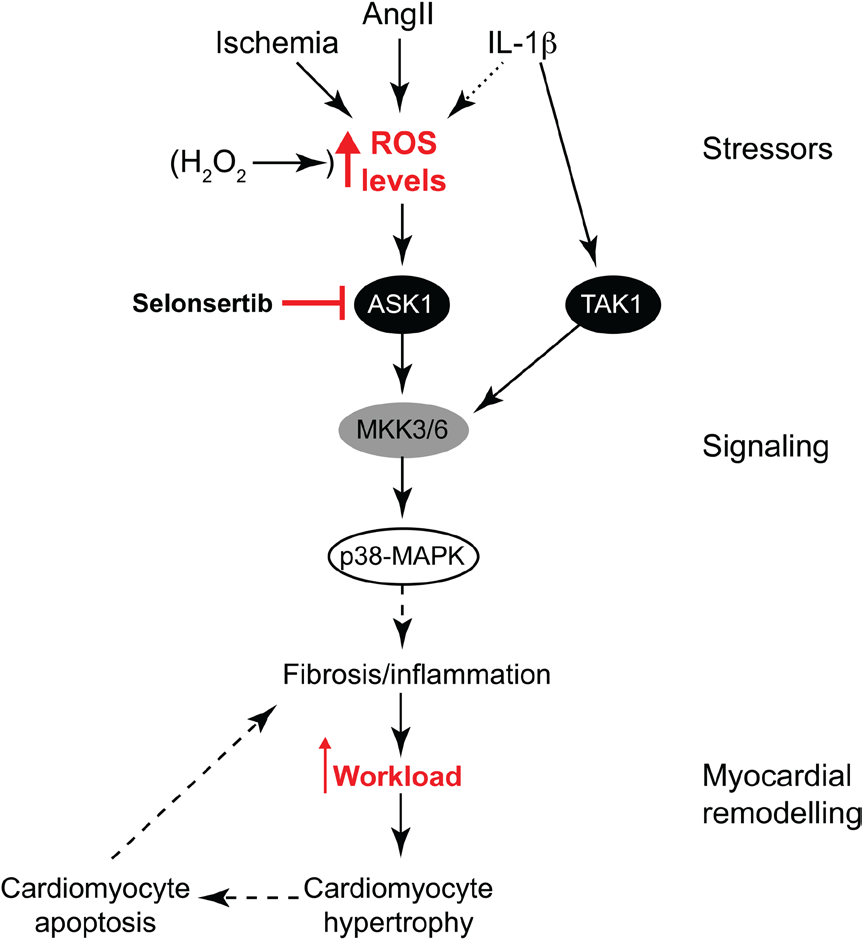
Schematic representation of ASK1 (apoptosis signal-regulating kinase 1) signaling in the heart. Cardiac cell stresses, such as ischemia and Ang II (angiotensin II), along with IL (interleukin)-1β to some extent, increase reactive oxygen species (ROS) signaling in the heart. These stressors or direct application of H_2_O_2_ lead to ASK1 activation. In contrast, redox signaling does not activate TAK1 (transforming growth factor–activated kinase 1), which is activated directly by IL-1β. In cardiomyocytes, ASK1 signals selectively to p38-MAPKs (mitogen-activated protein kinases), which, in the context of Ang II–induced hypertension in vivo, results in cardiac fibrosis and inflammation. This, in turn, increases cardiomyocyte workload, resulting in cardiomyocyte hypertrophy. Continued stress leads to cardiomyocyte apoptosis exacerbating cardiac fibrosis/inflammation. This destructive cycle of cardiac remodeling is broken by selonsertib, a selective ASK1 inhibitor. MKK indicates MAPK kinase.

Ang II–induced hypertension is associated with increased myocardial inflammation,^33^ although whether this precedes or is consequential to cardiac fibrosis remains to be defined. Ang II increased mRNA expression of proinflammatory cytokines (Figure S4) and inflammatory cell markers (Figure S5). Selonsertib did not affect upregulation of inflammatory cytokines but significantly reduced expression of myocarditis markers, reflective of protected myocardium. Therefore, our data strongly indicate that ASK1 operates as a profibrotic signal in the context of hypertension and is a potential therapeutic target to reduce pathological cardiac remodeling associated with this (Figure 6).

## Discussion

In recent years, ASK1 has emerged as a therapeutic target for fibrotic diseases, with ASK1 inhibitors in phase-III clinical trials for chronic kidney disease and nonalcoholic steatohepatitis.^16,17^ Here, we show that ASK1 inhibitors, such as selonsertib, have potential therapeutic value in hypertensive heart disease, reducing cardiac fibrosis together with cardiomyocyte hypertrophy (Figure 6). Thus, targeting the ASK1→p38-MAPK nexus has potential therapeutic viability as a treatment for hypertensive heart disease.

Our data with selonsertib are consistent with previous studies of mice with global ASK1 deletion subjected to a low dose of Ang II (0.288 mg/[kg·d]) in which cardiac hypertrophy and fibrosis were reduced.^31^ Our study used a higher hypertensive concentration of Ang II (0.8 mg/[kg·d]),^30^ resulting in greater fibrosis and hypertrophy and, still, selonsertib was effective at reducing both (Figures 4 and 5). Several studies indicate that ASK1 (as its name suggests) promotes cardiomyocyte death. Thus, global ASK1 knockout mice subjected to myocardial infarction or pressure-overload (models with much higher wall stresses than with Ang II–induced hypertension that is particularly associated with cardiomyocyte death) exhibit reduced cardiac hypertrophy and remodeling.^20^ Furthermore, cardiomyocyte overexpression of ASK1 increases cardiomyocyte apoptosis in response to stressors, exacerbating progression to heart failure in myocardial infarction and pressure-overload mouse models.^14^ ASK1 overexpression in cardiomyocytes does not promote hypertrophy in vivo per se,^14^ yet selonsertib inhibited cardiomyocyte hypertrophy in Ang II–induced hypertension (Figure 4). Here, it is important to consider the in vivo context taking into account the established profibrotic effects of ASK1. ASK1 inhibitors do not appear to affect blood pressure in humans (Selonsertib in adults with pulmonary arterial hypertension and Safety and Efficacy of Selonsertib in Adults With Nonalcoholic Steatohapetitis [NASH] and Bridging [F3] Fibrosis trials^16,17,34,35^) or in a mouse model of kidney fibrosis associated with hypertension,^36^ so it is unlikely that the reduction in cardiac fibrosis and cardiomyocyte hypertrophy with selonsertib seen in our studies with Ang II are due to an effect on blood pressure. It is more that the inhibition of fibrosis by selonsertib, associated with reduced paracrine signaling and inflammation, reduces cardiomyocyte workload, improving cardiomyocyte survival. The lower overall workload means that cardiomyocyte hypertrophy is also reduced.

As a ROS-sensing kinase, ASK1 is sensitive to an array of physiological and pathological processes and stimuli. Our data clearly support this because ASK1 is activated in cells in a time- and dose-dependent manner by direct redox challenge with H_2_O_2_ (Figures 1 and 2). However, ASK1 was activated by moderate levels of ROS (Figure 3) classically associated with apoptosis.^27^ In contrast, high levels of oxidative stress more associated with necrotic forms of cell death did not activate ASK1 which has a bell-shaped dose-response curve. In accord with this, ASK1 was activated only by ischemia (associated with moderate levels of ROS) in perfused hearts, but not on reperfusion (with much higher levels of ROS).^29^ Our data for ASK1 are consistent with previous studies of myocardial infarction in ASK1 knockout mice.^20^ Here, ASK1 deletion reduced the percentage of apoptotic cardiomyocytes particularly in the peri-infarct zone, a region likely to be subjected to lower ROS levels than cells in the infarct zone itself. It is difficult to correlate the levels of exogenously applied H_2_O_2_ with endogenous levels. However, IL-1β treated hearts exhibited an increase of ≈60 µmol/L H_2_O_2_ per mg of protein in perfused rat hearts (Figure 1C), so allowing for degradation of exogenous H_2_O_2_ as it enters cells, together with loss of endogenous H_2_O_2_ on production from the heart, the concentrations of H_2_O_2_ required to activate ASK1 (Figures 1 and 2) are in an appropriate range.

Other stimuli, although not activating ASK1 directly, may still activate ASK1 via secondary production of ROS. This appears to be the case with IL-1β which activated ASK1 in an intact perfused heart in which it increased oxidative stress (Figure 1). We could not detect activation of ASK1 in cardiomyocytes at any concentration of IL-1β that we studied (Figure S2). The reason is not clear, but the environment of cultured cardiomyocytes which have a good nutrient supply and efficient gas exchange contrasts with that of an intact beating heart in an ex vivo perfusion setting. The latter is under greater stress and subject to greater constraints with respect to oxygen supply and demand. This may deplete antioxidant reserves more rapidly and any small effect of IL-1β to increase ROS may be exaggerated to a sufficient degree that ASK1 may be activated. Other proinflammatory cytokines also activate ROS and stimulate ASK1 indirectly. For example, TNFα (tumor necrosis factor-α) signals through the ROS-generating NADPH oxidase, NADPH oxidase-1, to activate ASK1 in vascular smooth muscle cells.^37^ Proinflammatory cytokines alone do not promote apoptosis in primary neonatal cardiomyocytes,^38^ but the question remains of whether proinflammatory cytokines promote apoptosis via a ROS-ASK1 sensing system in compromised cells. The rheostat-like response of ASK1 to redox stress is in accord with our previous work showing that different degrees of oxidative stress differentially regulate signaling pathways and gene/protein expression.^24^ For ASK1, activation by moderate levels of ROS is likely to favor regulated (less damaging) forms of cell death such as apoptosis over unregulated necrotic cell death associated with a high level of inflammation.

ASK1 is implicated in activation of p38-MAPKs or JNKs in a plethora of studies in many different cells,^39^ raising a question of signal specificity. In cardiomyocytes and the heart overall, our data demonstrated that the signal from ASK1 is highly specific for p38-MAPK activation with little/no input into JNKs (Figures 2 and 3). In contrast, TAK1, as activated by IL-1β, activates both MAPK pathways (Figure 2; Figure S2), with cross-talk to ASK1 only in the intact heart consequential to an altered redox balance. One factor may be the hard-wiring of signaling networks in primary cells, particularly terminally differentiated cells, such as cardiomyocytes. However, although our data are consistent with previous studies in perfused hearts showing that ASK1 is activated in concert with p38-MAPKs,^15^ they contrast with other studies of the heart suggesting that ASK1 activates JNKs.^14,40^ It should be borne in mind that JNKs and p38-MAPKs are both stress-responsive, and interfering with one pathway via genetic modification may result in an increased stress that influences the other, making it difficult to dissect the signaling. For example, although cardiomyocyte ASK1 overexpression is associated with increased activation of JNKs (rather than p38-MAPKs) with pressure-overload over 1 to 8 weeks,^14^ this may be secondary to the remodeling response which includes enhanced fibrosis and inflammation. In our case, by using a small molecule inhibitor, we were able to assess the acute (ie, direct) signaling pathway.

Although there is much speculation and suggestion that ASK1 inhibitors may be useful for cardiac diseases,^16,17,21^ such drugs are not yet being assessed in clinical trials for this purpose. They have been tested in relation to nonalcoholic steatohepatitis, chronic kidney disease, and pulmonary hypertension in phase-II or phase-III trials,^16^ so they are clearly considered safe. Our data with selonsertib support the concept that reducing ASK1 activity in the heart is a desirable strategy for reducing fibrosis and thus the workload of the heart in disease states such as hypertension and is independent of a direct effect on blood pressure.^36^ Other studies with genetically modified mice indicate that ASK1 is also a strong therapeutic target in myocardial infarction in which inhibition of ASK1 may facilitate cardiomyocyte survival. A caveat is that because ASK1 is involved in fibrosis, inhibiting ASK1 may have a detrimental effect on establishing a stable fibrotic scar. It may be important, therefore, to tailor the therapy towards hypertension-associated cardiac dysfunction or other cardiac diseases with pronounced interstitial fibrosis.

## Perspective

Hypertensive heart disease is a poorly controlled and progressive condition associated with myocardial fibrosis, along with cardiomyocyte dysfunction and death, cumulating in cardiac stiffening and heart failure. This study demonstrates that the ROS-sensing enzyme ASK1 represents an ideal therapeutic target for hypertensive heart disease. ASK1 inhibitors are developed as antifibrotic therapies in other diseases and may be useful for hypertensive heart disease, reducing fibrosis and, therefore, improving cardiac function. At a mechanistic level, our data define a very specific ROS→ASK1→p38-MAPK signaling modality, suggesting that p38-MAPK(s) play a significant role in development of cardiac fibrosis. Clearly, other components of the pathway constitute alternative therapeutic targets. Thus, targeting the ASK1→p38-MAPK nexus in the heart has potential therapeutic viability as a treatment for hypertensive heart disease.

## Sources of Funding

This work was supported by the British Heart Foundation (grant numbers PG/13/71/30460, PG/17/11/32841, PG/15/24/31367, PG/15/31/31393, FS/18/33/33621, FS/19/24/34262) and the Wellcome Trust Institutional Strategic Support Fund (204809/Z/16/Z).

## Acknowledgments

D.N. Meijles and A. Clerk conceived and designed the experiments. D.N. Meijles, J.J. Cull, T. Markou, S.T.E. Cooper, Z.H.R. Haines, S.J. Fuller, P. O’Gara, and A. Clerk performed the experiments. D.N. Meijles, T. Markou, M.N. Sheppard, S.E. Harding, P.H. Sugden, and A. Clerk analyzed and interpreted the data. D.N. Meijles, P.H. Sugden, and A.Clerk drafted and edited the article.

## Disclosures

None.

## Supplementary Material



## Novelty and Significance

### What Is New?

- We established that reactive oxygen species signals through ASK1 (apoptosis signal-regulating kinase 1) exclusively to p38-MAPK (mitogen-activated protein kinases) in the heart, accounting for activation of p38-MAPK in ischemia. Inhibiting ASK1 reduced cardiac fibrosis in hypertension-induced cardiac hypertrophy in mice in vivo.

### What Is Relevant?

- Because cardiac fibrosis predisposes to heart failure, ASK1 inhibitors (in clinical trials for other fibrosis-associated diseases) may be therapeutically useful for hypertension-associated cardiac hypertrophy, reducing or preventing progression to heart failure in humans.

### Summary

Our data establish that a reactive oxygen species-ASK1-p38-MAPK signal operates in the heart and, in the context of hypertension, this is linked to increased cardiac fibrosis. Targeting ASK1 with existing small molecule inhibitors may be beneficial in hypertensive heart disease, reducing cardiac fibrosis and, thus, maintaining cardiac function.
