# Supplementary material for: Redox Regulation of Cardiac ASK1 (Apoptosis Signal-Regulating Kinase 1) Controls p38-MAPK (Mitogen-Activated Protein Kinase) and Orchestrates Cardiac Remodeling to Hypertension
Source: Hypertension. 2020 Sep 9;76(4):1208–18. doi: 10.1161/HYPERTENSIONAHA.119.14556 (PMC7480944; doi:10.1161/HYPERTENSIONAHA.119.14556)
Supplement: Supplementary file 1 [file hyp-76-1208-s001.docx]

**Redox regulation of cardiac ASK1 controls p38-MAPK and orchestrates cardiac remodelling to hypertension.**

Daniel N. Meijles^1,2^*, Joshua J. Cull^2^, Thomais Markou^2^, Susanna T.E. Cooper^1^, Zoe H.R. Haines^1^, Stephen J. Fuller^2^, Peter O’Gara^4^, Mary N. Sheppard^3^, Sian E. Harding^4^, Peter H. Sugden^2^, & Angela Clerk^2^*.

^1^Molecular and Clinical Sciences Institute, St George's University of London, London SW17 0RE, UK. ^2^School of Biological Sciences, University of Reading, Reading RG6 6AS, UK. ^3^CRY Cardiovascular Pathology Department, St. George's University of London, London, UK; St. George's Healthcare NHS Trust, London, SW17 0RE, UK. ^4^National Heart and Lung Institute, Faculty of Medicine, Imperial College London, London W12 0NN

**Online Materials and methods** (complete)

*Agonists and inhibitors*

Recombinant rat IL-1β was from R&D systems and dissolved in 0.05% (w/v) bovine serum albumin (BSA). H_2_O_2_ and N-acetyl cysteine (NAC) were from Sigma-Aldrich. Selonsertib was from Selleck Chemicals.

*Animals*

Animals were housed at the BioResource Unit at University of Reading (UK registered with a Home Office certificate of designation). All procedures were performed in accordance with UK regulations and the European Community Directive 86/609/EEC for animal experiments. Work was undertaken in accordance with local institutional animal care committee procedures (University of Reading) and the U.K. Animals (Scientific Procedures) Act 1986. Sprague-Dawley female rats with 2 d neonates, adult male Sprague-Dawley rats and wild-type C57Bl/6J mice (8 wks) were purchased from Charles River (UK) and transported to the BioResource Unit at University of Reading. Adult mice and rats were allowed to acclimatize for at least 7 d prior to experiments.

Mice were housed in Tecniplast IVC cages (total area 512 cm^2^; maximum 5 mice per cage). Adult male rats were housed in open top NKP cages (total area 1632cm^2^; maximum 5 rats per cage). Cages were supplied with aspen sawdust bedding, sizzle nesting, cardboard tunnels and housing. Additional enrichment included chew sticks and millet to encourage foraging behaviour. Animals were provided with water and food (SDS Rm3 pelleted food for mice; SDS RM3 expanded pelleted food for rats) *ad libitum*, with a 12:12 light/dark cycle and room temperature of 21°C. Neonatal rats were culled on arrival by schedule 1 (cervical dislocation) for which additional approval and licences are not required according to UK regulations. All animals were checked at least once a day by a trained, competent person and licence holders informed of any welfare issues, with consultation with a Named Veterinary Surgeon when necessary. Mice undergoing procedures were monitored using a score sheet and routinely culled if they reached a predefined endpoint agreed with the Named Veterinary Surgeon. Weights were taken before, during and at the end of the procedures. Mouse weights from the start and end of procedures are provided in **Table S2**. Mice were excluded after randomisation only if there was a health problem.

*AngII model and drug delivery in mice*

All drug delivery was achieved using Alzet osmotic pumps (model 1002), filled according to the manufacturer’s instructions in a laminar flow hood using sterile technique. Wild-type C57BL/6J male mice (at 10 - 12 wks) received either vehicle (acidified PBS) or angiotensin II (AngII, 0.8 mg/kd/d), plus selonsertib (4.0 mg/kg/d) dissolved in DMSO/PEG mix [50% (v/v) DMSO, 20% (v/v) polyethylene glycol 400, 5% (v/v) propylene glycol, 0.5% (v/v) Tween 80] or DMSO/PEG alone (vehicle) for 7 d. Minipumps were incubated overnight in sterile PBS (37°C) prior to implantation. Implantation was performed under continuous inhalation anaesthesia using isoflurane (induction at 5%, maintenance at 2 - 2.5%) mixed with 2 l/min O_2_. A 1 cm incision was made in the mid-scapular region and mice were given 0.05 mg/kg (s.c.) buprenorphine (Vetergesic, Ceva Animal Health Ltd.) to repress post-surgical discomfort. Minipumps were implanted portal first in a pocket created in the left flank region of the mouse. Wound closure used a simple interrupted suture with polypropylene 4-0 thread (Prolene, Ethicon). Mice were recovered singly and returned to their home cage once fully recovered.

A total of 40 mice were distributed between controls (n=10), AngII only (n=11), selonsertib only (n=8) or selonsertib plus AngII (n=11). The study was treated as exploratory and therefore correction for multiple comparisons was not applicable. Male mice were used to minimise animal numbers selecting for the sex with least variation. As an exploratory study for the effects of selonsertib on the cardiac response to AngII, groups sizes were estimated based on studies of other protein kinase inhibitors using a 5% type I error rate and power of 0.9. Weights were taken before, during and at the end of the procedures. Mouse weights from the start (day of minipump insertion) and end (day of final echocardiogram and harvest) of procedures are provided in **Table S2**. Allocation of mice to specific groups was on a random basis with randomization performed independently of the individual leading the experiment. Individuals conducting the *in vivo* studies were not blinded to experimental conditions for welfare monitoring purposes. Data and sample analysis (e.g. echocardiography, histology) was performed by individuals who were blinded to intervention.

*Cardiac ultrasound.*

Echocardiography was performed on anaesthetised mice using a Vevo 2100 imaging system equipped with a MS400 18-38 MHz transducer (Visualsonics). Mice were anaesthetised in an induction chamber with isoflurane (5% flow rate) with 1 l/min O_2_ then transferred to the heated Vevo Imaging Station. Anaesthesia was maintained with 1.5% isoflurane delivered via a nose cone. Left ventricular cardiac function and structure was assessed from short axis M-mode (all other measurements) images with the axis placed at the mid-level of the left ventricle at the level of the papillary muscles. Baseline scans were taken prior to experimentation (-7 to -3 days). Further scans were taken at 3 or 7 d following minipump implantation. Imaging was completed within 20 min. Mice were recovered singly and transferred to the home cage once fully recovered. Data analysis (Vevo LAB version 1.7.1) was performed by independent assessors blinded to intervention. Data were gathered from two scans taken from each time point, taking mean values across 5 cardiac cycles for each scan.

*Tissue harvesting and processing.*

Mice were culled by schedule 1 (CO_2_ followed by cervical dislocation). Hearts were excised quickly, washed in PBS, and snap-frozen in liquid N_2_ or fixed for histology.

*Histology*

Histological analysis was performed on mouse hearts subjected to *in situ* perfusion fixation with 10% formalin. Following immersion in 70% ethanol, hearts were embedded in paraffin, sectioned at 10 μm and stained using kits for either hematoxylin and eosin (H&E, Sigma) and Masson's trichrome (Polysciences) or picrosirius red for investigation of overall cardiac morphology and fibrosis, respectively, following the manufacturer’s instructions. For picrosirius red staining, sections were submerged in Weigert’s hematoxylin, washed, stained in picrosirius red (1 g/L Sirus red in saturated aqueous picric acid, 60 min), and differentiated in 0.5% acetic acid.

All heart sections were captured and stored digitally using a Nikon slide scanner. For analysis of myocyte cross‐sectional area, cells stained by H&E within the LV (excluding endocardial regions) were chosen at random and outline traced using the NDP.view2 software (Hamamatsu). Only cells with a single nucleus were included in the analysis. For assessment of fibrosis, 20X Massons-trichrome images of the entire LV were exported and the collagen fraction calculated as the ratio between the sum of the total area of fibrosis (blue colour) to the sum of the total tissue area (including the myocyte area) for the entire image using Image-J and expressed as a percentage. For investigating the pattern of LV fibrosis, images were also sub-divided between those which including vessels (for perivascular fibrosis) and those void of vessels (for interstitial fibrosis). All histological and data analysis was performed by independent assessors blinded to treatment groups.

*Adult rat heart perfusions*

Adult male (300-350 g) Sprague–Dawley rats were anaesthetised with a lethal intraperitoneal dose of Euthatal (pentobarbital sodium, 60 mg/kg), perfused i.v. with 1000 units/kg of heparin (1000 units/ml), and the heart and lungs removed into modified ice-cold KHBBS (25 mM NaHCO_3_, 119 mM NaCl, 35 mM KCl, 2.5 mM CaCl_2_, 1.2 mM MgSO_4_, 1.2mM KH_2_PO_4_ equilibrated with 95% O_2_/5% CO_2_) whilst the heart was still beating. Next, hearts were cleaned of surrounding tissues before aortic cannulation and perfusion with equilibration buffers as published.^1^ For all studies except those with N-acetyl cysteine (NAC), hearts were perfused retrogradely (37°C at 70 mmHg) as described previously with a 15 min equilibration period. For studies with NAC, hearts were perfused at constant flow (6-8 ml/min/g tissue). At the end of the equilibration period, hearts were perfused with 1 mM H_2_O_2_ or 25 ng/ml IL-1β. Alternatively, global ischaemia was imposed by clamping of the perfusion line (15 min) without or with reperfusion (30 min). In experiments using ROS scavengers or ASK1 inhibitors, NAC (4 mM), selonsertib (1 µM) or vehicle (0.1% (v/v) DMSO) was included in the equilibration buffer. In all studies, controls were conducted in which perfusion was continued with the equilibration buffer for the same duration. At the end of each study protocol, hearts were ‘freeze-clamped’ and pulverized under liquid nitrogen in a pestle and mortar. The powders were stored at −80°C for subsequent biochemical analysis (see below).

*Cell cultures and treatments*

Neonatal rat ventricular myocytes (NRVMs) were prepared and cultured from 2-4 d Sprague-Dawley rats as previously described,^2^ with minor modification. Ventricles were dissected and dissociated by serial digestion at 37°C with 6800 U Type II Worthington collagenase (supplied by Lonza) and 0.6 mg/ml pancreatin (Sigma-Aldrich, cat. No. P3292) in sterile digestion buffer (116 mM NaCl, 20 mM HEPES, 0.8 mM Na_2_HPO_4_, 5.6 mM glucose, 5.4 mM KCl and 0.8 mM MgSO_4_, pH 7.35). The first digestion supernatant (5 min, 37°C, 160 cycles/min in a shaking waterbath) was discarded. Cell suspensions from subsequent digestions (4×25 min, 37°C 136 cycles/min shaking) were recovered by centrifugation (5 min, 60×g) and the cell pellet resuspended in plating medium (Dulbecco's modified Eagle's medium (DMEM)/medium 199 [4:1 (v/v)] containing 15% (v/v) fetal calf serum (FCS) and 100 units/ml penicillin and streptomycin). Cells were pre-plated on plastic tissue culture dishes (30 min) to remove cardiac non-myocytes; non-adherent cardiomyocytes were collected and viable cells counted by Trypan Blue (Sigma-Aldrich UK) exclusion using a haemocytometer. For immunoblotting or qPCR, viable cardiomyocytes were plated at a density of 4×10^6^ cells/dish on 60 mm Primaria dishes pre-coated with sterile 1% (w/v) gelatin (Sigma-Aldrich UK). After 18 h, myocytes were beating spontaneously. For all experiments, the plating medium was withdrawn after 18 h and cells were incubated in serum-free maintenance medium (DMEM/medium [4:1 (v/v)] containing 100 units/ml penicillin and streptomycin) for a further 24 h.

Human cardiac fibroblasts (HCFs) were purchased from PromoCell (donor lot#: 437Z012.4) and cultured as recommended in fibroblast growth medium-3. Cells were seeded into 6-well plates the day before experimentation in serum-reduced (0.1% v/v) growth medium.

Cells were subjected to vehicle, or H_2_O_2_ (0-10 μM) or IL-1β (0-25 ng/ml) for the times and at the concentrations indicated. In experiments with selonsertib, cells were pre-incubated for 15 min prior to addition of H_2_O_2_ or IL-1β. Agonists/inhibitor were added directly to the tissue culture medium.

*Immunoblotting*

For analysis of protein kinases in cultured cells, cells were washed with ice-cold PBS and scraped into 150 µl glycerophosphate buffer [20 mM β-glycerophosphate (pH 7.5), 50 mM NaF, 2 mM EDTA, 0.004 mM microcystin LR, 1% (v/v) Triton X-100, 5 mM dithiothreitol, 10 mM benzamidine, 0.2 mM leupeptin, 0.01 mM trans-epoxy succinyl-l-leucylamido-(4-guanidino)butane, 0.3 mM phenylmethylsulfonyl fluoride]. For analysis of protein kinases in rat or mouse heart powders, samples were extracted in 6 vol glycerophosphate buffer (relative to weight of powder), vortexed and extracted on ice (10 min). Extracts were centrifuged (10,000×g, 4°C) for 5 or 10 min for cardiomyocytes or heart powders, respectively. The supernatants were removed, a sample was taken for protein assay and the remainder boiled with 0.33 vol SDS-polyacrylamide gel electrophoresis (SDS-PAGE) sample buffer (0.33 M Tris-HCl pH 6.8, 10% (w/v) SDS, 13% (v/v) glycerol, 133 mM dithiothreitol, 0.2 mg/mL bromophenol blue). Protein concentrations were determined by BioRad Bradford assay using BSA standards. Proteins were separated by SDS-PAGE on 10% (for TAK1, p38-MAPK, JNK) or 8% (for ASK1) (w/v) polyacrylamide mini-gels and transferred electrophoretically to nitrocellulose using a BioRad semi-dry transfer cell (10V, 60 min). Proteins were detected as previously described^3^ using primary antibodies as indicated in Table S1. Bands were detected by enhanced chemiluminescence using ECL Prime Western Blotting detection reagents with visualisation using an ImageQuant LAS4000 system (GE Healthcare). ImageQuant 7.0 software (GE Healthcare) was used for densitometric analysis of the bands.

For analysis of Collagen 1a1, heart powders were extracted in glycerophosphate buffer plus inhibitors as described above and centrifuged (10,000×g, 4°C, 10 min). The pellets were resuspended in 8 vol Buffer B (10 mM HEPES, pH 7.9, 400 mM KCl, 1.5 mM MgCl_2_, 0.3 mM Na_3_VO_4_ 5 mM dithiothreitol) containing protease and phosphatase inhibitors) to remove contractile proteins. Samples were vortexed, incubated on ice (10 min) and then centrifuged (10,000 × g, 10 min, 4°C). The pellets were further extracted in 8 vol SDS-PAGE sample buffer diluted 1:1 with Buffer A without bromophenol blue. Samples were boiled for 5 min and centrifuged (12,000 × g, 10 min, 4°C). The supernatants were used to assess collagen content. Protein concentrations were determined using a BCA protein assay (Thermo Scientific). Remaining samples were boiled with 0.33 volumes of diluted (1:1) SDS-PAGE sample buffer containing bromophenol blue.

*ROS measurement*

Cardiac H_2_O_2_ production was measured using the Amplex Red (Invitrogen Inc.) assay, as described previously,^4^ using tissue homogenates. Briefly, protein (50 μg/ml) was added to the wells on a 96-well black plate containing the assay mixture (25 mM Hepes pH 7.4, 0.12 M NaCl, 3 mM KCl, 1 mM MgCl_2_, 0.1 mM Amplex Red, 0.32 U/ml horseradish peroxidase). The reaction was initiated by the addition of 36 μM NADPH, as published previously.^4^ Fluorescence was detected using the BMG FLUOstar Multi-Mode Microplate Reader with a 530/25 excitation and a 590/35 emission filter. The reaction was monitored for 60 min at 30°C; the change in emission intensity was linear during this interval. To confirm the H_2_O_2_ signal, catalase (300 U/ml) was added in parallel wells, and the catalase-inhibitable rate of H_2_O_2_ production was quantified from an H_2_O_2_ standard curve.

*RNA preparation and qPCR*

Total RNA was prepared using RNA Bee (AMS Biotechnology Ltd) with 1 ml per 10-15 mg mouse heart powder. RNA was prepared according to the manufacturer's instructions. RNA was dissolved in nuclease-free water and purity assessed from the A_260_/A_280_ measured using an Implen NanoPhotometer (values of 1.8–2.1 were considered acceptable). RNA concentrations were determined from the A_260_ values. Quantitative PCR (qPCR) analysis was performed as previously described.^5^ Total RNA (2 µg) was reverse transcribed to cDNA using High Capacity cDNA Reverse Transcription Kits with random primers (Applied Biosystems) according to the manufacturer's instructions. qPCR was performed using an ABI Real-Time PCR 7500 system (Applied Biosystems). Optical 96-well reaction plates were used with iTaq Universal SYBR Green Supermix (Bio-Rad Laboratories Inc.) according to the manufacturer’s instructions. Primers were from PrimerDesign or ThermoFisher (Table S3). Results were normalized to the housekeeping gene *Gapdh*, and relative quantification was obtained using the ΔCt (threshold cycle) method; relative expression was calculated as 2^−ΔΔCt^, and normalised to vehicle controls.

*Statistical analysis*

Data are the means ± SEM as detailed in the figure legends with individual data points provided where appropriate. Data were collected and analysed in Microsoft Excel, with statistical analysis performed in GraphPad Prism 7.0 using a *t*-test, one-way ANOVA or two-way ANOVA as indicated. A multiple comparison test (Holm-Sidak's) was used in combination with ANOVA. *P*<0.05 was considered statistically significant.

**Full method references**

1. Clerk A, Fuller SJ, Michael A and Sugden PH. Stimulation of "stress-regulated" mitogen-activated protein kinases (stress-activated protein kinases/c-Jun N-terminal kinases and p38-mitogen-activated protein kinases) in perfused rat hearts by oxidative and other stresses. *J Biol Chem*. 1998;273:7228-34.

2. Marshall AK, Barrett OP, Cullingford TE, Shanmugasundram A, Sugden PH and Clerk A. ERK1/2 signaling dominates over RhoA signaling in regulating early changes in RNA expression induced by endothelin-1 in neonatal rat cardiomyocytes. *PLoS One*. 2010;5:e10027.

3. Meijles DN, Zoumpoulidou G, Markou T, Rostron KA, Patel R, Lay K, Handa BS, Wong B, Sugden PH and Clerk A. The cardiomyocyte "redox rheostat": Redox signalling via the AMPK-mTOR axis and regulation of gene and protein expression balancing survival and death. *J Mol Cell Cardiol*. 2019;129:118-129.

4. Meijles DN, Sahoo S, Al Ghouleh I, Amaral JH, Bienes-Martinez R, Knupp HE, Attaran S, Sembrat JC, Nouraie SM, Rojas MM, Novelli EM, Gladwin MT, Isenberg JS, Cifuentes-Pagano E and Pagano PJ. The matricellular protein TSP1 promotes human and mouse endothelial cell senescence through CD47 and Nox1. *Sci Signal*. 2017;10.

5. Fuller SJ, Osborne SA, Leonard SJ, Hardyman MA, Vaniotis G, Allen BG, Sugden PH and Clerk A. Cardiac protein kinases: the cardiomyocyte kinome and differential kinase expression in human failing hearts. *Cardiovasc Res*. 2015;108:87-98.

**Supplemental Table S1.** **Antibodies used for immunoblotting.** Primary antibodies from CST (Cell Signaling Technologies) or Sigma, secondary antibodies from Dako.

| **Protein** | **Source** | **Cat. no.** | **Host** | **Dilution** |
| --- | --- | --- | --- | --- |
| Phospho-ASK1(T845) | CST | 3765 | Rabbit | 1/850 |
| Phospho-ASK1(S967) | CST | 3764 | Rabbit | 1/850 |
| Total ASK1 | CST | 3762 | Rabbit | 1/850 |
| Phospho-TAK1(T184/187) | CST | 4508 | Rabbit | 1/1000 |
| Total TAK1 | CST | 5206 | Rabbit | 1/1000 |
| Phospho-p38-MAPK (T182/Y182) | CST | 4511 | Rabbit | 1/1000 |
| Total p38-MAPK | CST | 8690 | Rabbit | 1/1000 |
| Phospho-JNKs (T183/Y185) | CST | 4668 | Rabbit | 1/1000 |
| Total JNKs | CST | 9252 | Rabbit | 1/1000 |
| Phospho-ERK1/2 (T202/Y204) | CST | 4370 | Rabbit | 1/1000 |
| β-actin | Sigma | A1978 | Mouse | 1/1000 |
| Collagen-1A1 | CST | 84336 | Rabbit | 1/1000 |
| Anti-Rabbit immunoglobulins/HRP | Dako | P0448 | Goat | 1/5000 |
| Anti-Mouse immunoglobulins/HRP | Dako | P0260 | Rabbit | 1/5000 |

**Supplemental Table S2. Mouse body weights (g).** *p <0.05 relative to starting weight. ^#^p <0.05 vs vehicle.

|  | **Start** | | | **End** | | |
| --- | --- | --- | --- | --- | --- | --- |
|  | **Mean** | **SEM** | **n** | **Mean** | **SEM** | **n** |
| **Vehicle** | 27.08 | 0.70 | 10 | 27.56 | 0.71 | 10 |
| **Selonsertib** | 26.95 | 0.34 | 8 | 27.89 | 0.20 | 8 |
| **AngII** | 26.36 | 0.53 | 11 | 26.45 | 0.51 | 11 |
| **Selonsertib + AngII** | 25.19 | 0.66 | 11 | 25.21 | 0.56 | 11 |

**Supplemental Table S3.** Mouse Custom qPCR primers from PrimerDesign (PD) or ThermoFisher (TF).

| **Gene Symbol** | **Source** | **Sense Primer (5'→3')** | **Antisense Primer (5'→3')** |
| --- | --- | --- | --- |
| *Col1a1* | PD | TCGTGGCTTCTCTGGTCTC | CCGTTGAGTCCGTCTTTGC |
| *Col2a1* | TF | ACTGGTAAGTGGGGCAAGAC | CCACACCAAATTCCTGTTCA |
| *Col3a1* | TF | GGAACCTGGTTTCTTCTCACC | TAGGACTGACCAAGGTGGCT |
| *Col4a1* | TF | CTGGCACAAAAGGGACGAG | ACGTGGCCGAGAATTTCACC |
| *Ddr2* | PD | GCACTTGGTGAATTAATTAGAATCCTG | GGACAACTAAATGGTCCCTCCC |
| *Gapdh* | TF | TCACCACCATGGAGAAGGC | GCTAAGCAGTTGGTGGTGCA |
| *IL11* | PD | TGACGGAGATCACAGTCTGGA | CGGAGGTAGGACATCAAGTCTAC |
| *Lox* | PD | GACATTCGCTACACAGGACAT | AACACCAGGTACGGCTTTATC |
| *Myh6* | PD | GAGATCGAGGACCTGATGG | TCATACTTCTGCTTCCACTCA |
| *Myh7* | PD | GAGATCGAGGACCTGATGG | TCATACTTCTGCTTCCACTCA |
| *Nppa* | PD | GATGGATTTCAAGAACCTGCTAGA | CTTCCTCAGTCTGCTCACTCA |
| *Nppb* | PD | TCCAGCAGAGACCTCAAAATTC | CAGTGCGTTACAGCCCAAA |
| *Post1* | TF | TTCCTCTCCTGCCCTTATATGC | CCTGATCCCGACCCCTGAT |
| *Tgfb* | TF | GGAGAGCCCTGGATACCAAC | CAACCCAGGTCCTTCCTAAA |
| *Thbs1* | TF | GCGTTGCCAGGCTCCGAGTT | GGTGCGCAGGCCCTTCAGTT |
| *Timp1* | TF | TACGCCTACACCCCAGTCAT | GCCCGTGATGAGAAACTCTTC |
