# Supplementary figures and images for: Redox Regulation of Cardiac ASK1 (Apoptosis Signal-Regulating Kinase 1) Controls p38-MAPK (Mitogen-Activated Protein Kinase) and Orchestrates Cardiac Remodeling to Hypertension
Source: Hypertension. 2020 Sep 9;76(4):1208–18. doi: 10.1161/HYPERTENSIONAHA.119.14556 (PMC7480944; doi:10.1161/HYPERTENSIONAHA.119.14556)

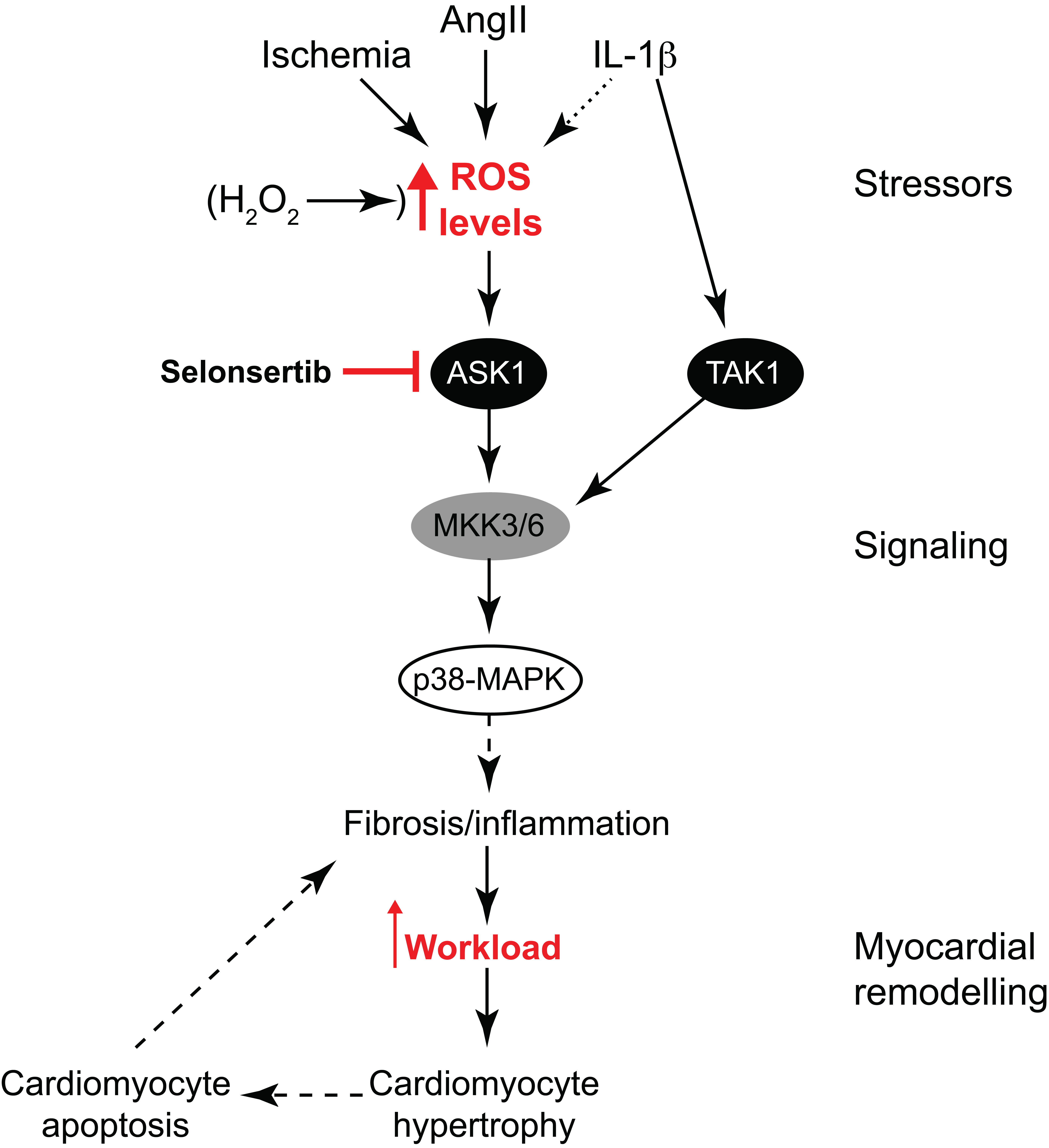

Supplement: Supplementary file 2 [file hyp-76-1208-s002.jpg]
