# Supplementary material for: Redox Regulation of Cardiac ASK1 (Apoptosis Signal-Regulating Kinase 1) Controls p38-MAPK (Mitogen-Activated Protein Kinase) and Orchestrates Cardiac Remodeling to Hypertension
Source: Hypertension. 2020 Sep 9;76(4):1208–18. doi: 10.1161/HYPERTENSIONAHA.119.14556 (PMC7480944; doi:10.1161/HYPERTENSIONAHA.119.14556)
Supplement: Supplementary file 3 [file hyp-76-1208-s003.pdf]

*** Short In Vivo Checklist**

AHA - Preclinical Animal Testing: Prevention of bias is important for experimental cardiovascular research. ***This short checklist must be completed, and the answers should be clearly presented in the manuscript as well.*** *The checklist will be used by reviewers and editors but will not be published. If a revision is requested, you will be required to complete at revision submission a more detailed checklist that will be published with the accepted article.*

This study involves animals:
Yes

Animals

Species, age, sex, strains, and sources of animals are described:	Yes

Randomization

Randomization and allocation concealment were performed:	Yes

Blinding

Blinding was performed:	Yes

Inclusions and Exclusions (a)

Specific criteria for inclusions and exclusions are specified:	Yes

Inclusions and Exclusions (b)

Criteria for inclusions and exclusions were set before the study:	Yes

Reporting of Excluded Animals

All animals excluded after the randomization are reported:	Yes

Statistical Methods

Statistical Methods are described:	Yes

Date completed: 07/22/2020 07:59:50
User pid: 195261
